# Supplementary material for: Targeting E2F Sensitizes Prostate Cancer Cells to Drug-Induced Replication Stress by Promoting Unscheduled CDK1 Activity
Source: Cancers (Basel). 2022 Oct 10;14(19):4952. doi: 10.3390/cancers14194952 (PMC9564059; doi:10.3390/cancers14194952)
Supplement: Supplementary file 1 [file cancers-14-04952-s001.zip › Supplementary Figures.pdf]

Targeting E2F Sensitizes Prostate Cancer Cells to Drug-Induced Replication Stress by Promoting Unscheduled CDK1 Activity

Mohaddase Hamidi, Ainhoa Eriz, Jone Mitxelena, Larraitz Fernandez-Ares, Igor Aurrekoetxea , Patricia Aspichueta, Ainhoa Iglesias-Ara and Ana M. Zubiaga

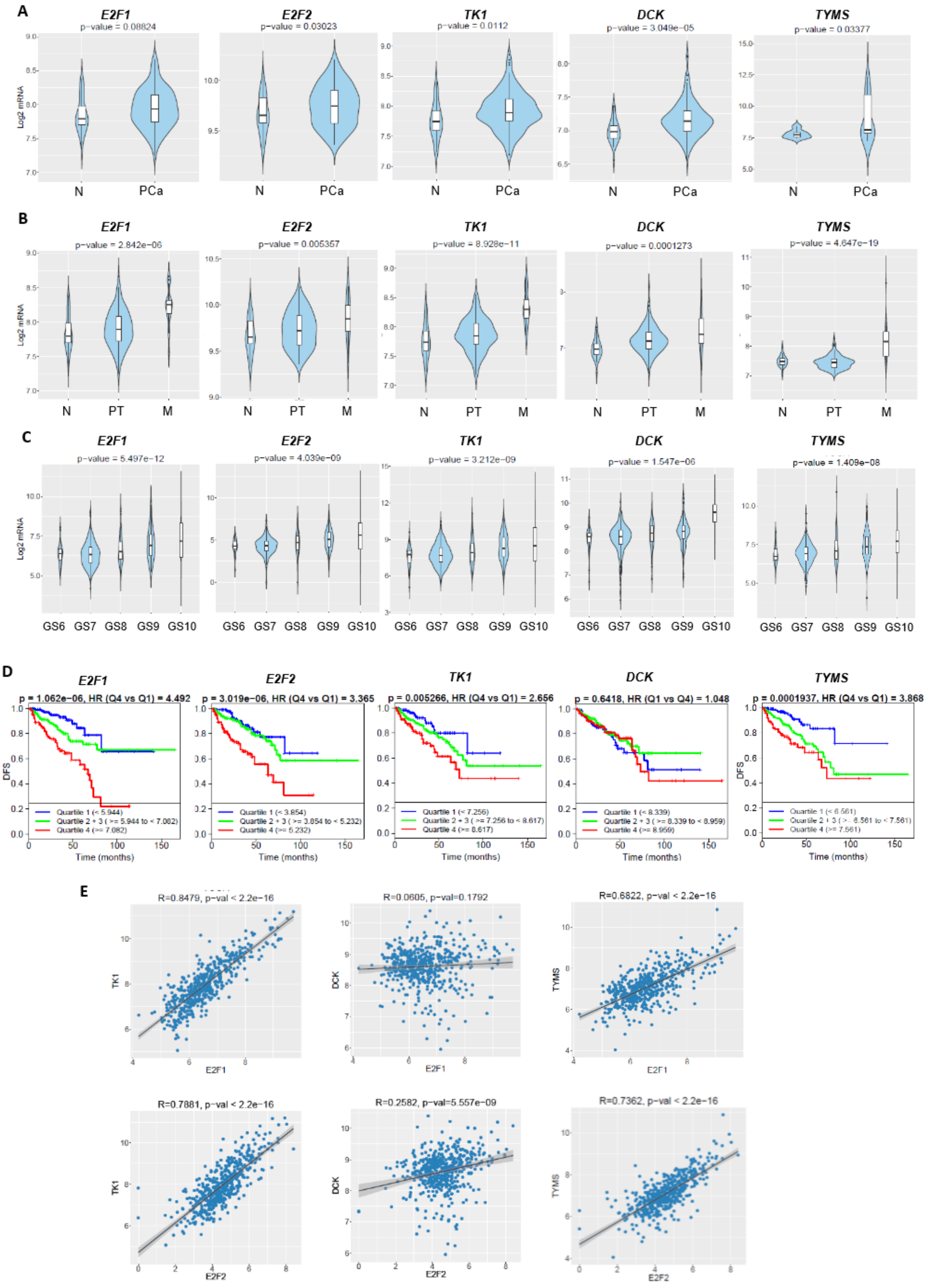

<u>Supplementary Figure S1</u>. Increased expression of E2F1, E2F2, TK1, DCK1 and TYMS in prostate cancer vs. normal tissue correlates with worse prognosis and shorter disease-free survival. **A)**

Violin plots depicting the expression of the gene of interest between non-tumoral (N) and prostate cancer specimens (PC) in the Taylor (n=176) dataset. The Y-axis represents the Log2-normalized gene expression (fluorescence intensity values for microarray data or, sequencing reads values obtained after gene quantification with RSEM and normalization using Upper Quartile in case of RNAseq). A Student T-test is performed in order to compare the mean gene expression between two groups. **B)** Violin plots depicting the expression of the gene of interest among non-tumoral (N), primary tumor (PT) and metastatic (M) PC specimens in the Taylor (n=176) dataset. The Y-axis represents the Log2-normalized gene expression (fluorescence intensity values for microarray data or, sequencing reads values obtained after gene quantification with RSEM and normalization using Upper Quartile in case of RNAseq). An ANOVA test is performed in order to compare the mean gene expression among two groups. **C)** Violin plots depicting the expression of the indicated gene among PC specimens of the indicated Gleason grade in the TCGA (n=496) dataset. The Y-axis represents the Log2-normalized gene expression (fluorescence intensity values for microarray data or, sequencing reads values obtained after gene quantification with RSEM and normalization using Upper Quartile in case of RNAseq). Gleason grade is indicated as GS6, GS7, GS8, GS8+9, GS9, GS10. An ANOVA test is performed in order to compare the mean among groups. **D)** Kaplan-Meier curves representing the disease-free survival (DFS) of patient groups selected according to the quartile expression of the indicated gene in the TCGA (n=496) dataset. Quartiles represent ranges of expression that divide the set of values into quarters. Quartile color code: Q1 (Blue), Q2 + Q3 (Green), Q4 (Red). Each curve represents the percentage (Y-axis) of the population that exhibits recurrence of the disease along time (X-axis, in months) for a given gene expression distribution quartile. Vertical ticks indicate censored patients. A Mantel-Cox test is performed in order to compare the differences between curves, while a Cox proportional hazards regression model is performed to calculate de Hazard Ratio (HR) between the indicated groups. **E)** Plotted values correspond to the log2-normalized gene expression values (fluorescence intensity or RSEM-UQ) for two genes (in X and Y-axis) for each patient in the

TCGA (n=496) dataset. Black line represents linear regression, grey area indicates the limits of the confidence intervals and R and p indicate Pearson´s correlation coefficient and statistical significance, respectively.

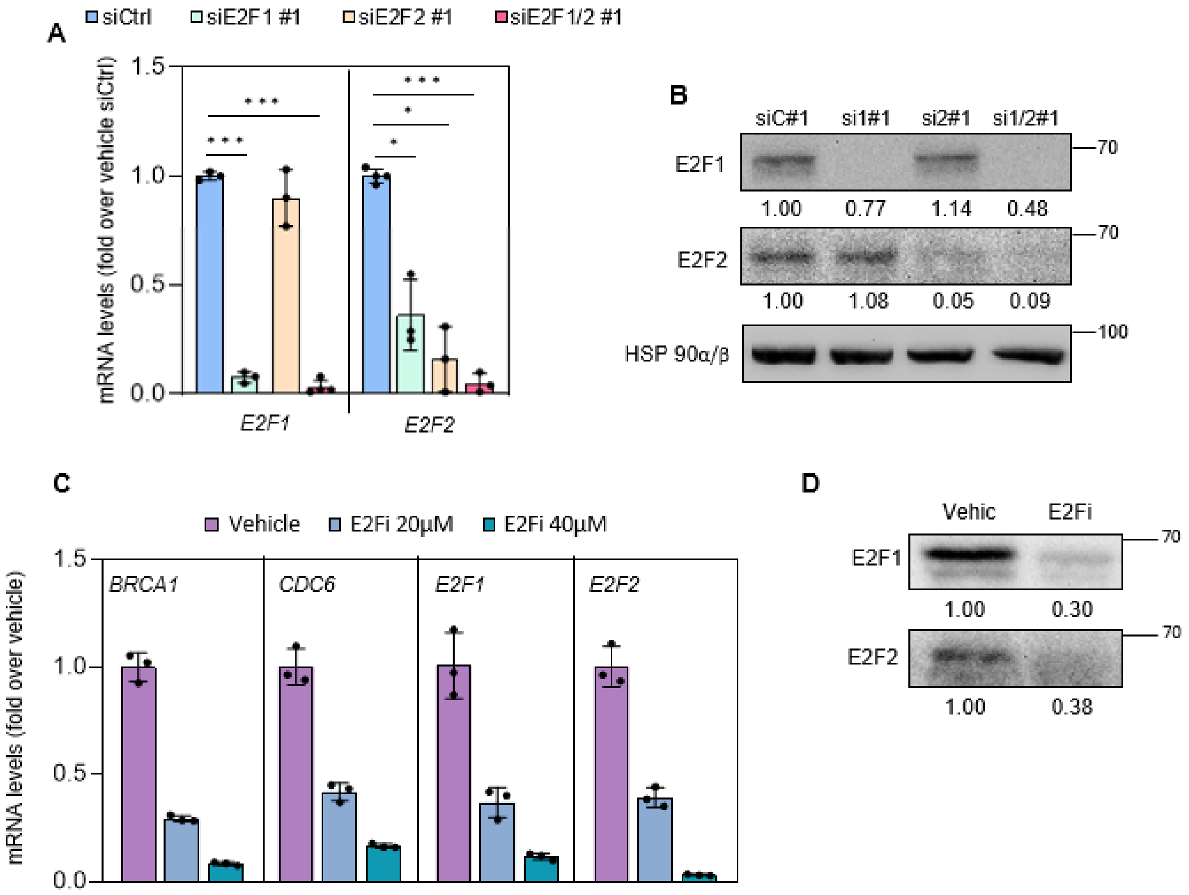

Supplementary Figure S2. **A,B)** Silencing efficiency of set #1 siRNAs for E2F1 and E2F2. **A)** RT-qPCR analysis of E2F1 and E2F2 in PC3 cells 96h after transfection with non-target control siRNAs (siCtrl), siRNAs specific for E2F1 (siE2F1#1), for E2F2 (siE2F2#1) or with their combination (siE2F1/2#1). *L19* was used as normalization control. Results are expressed as fold-over vehicle siCtrl sample (mean ± SD) from 3 independent experiments. *** $p < 0.0001$, ** $p < 0.001$, * $p < 0.05$. **B)** Representative Western blot analysis of E2F1 and E2F2 in extracts prepared as previously detailed. Expression of HSP90 was used as loading control. Numbers below the bands correspond to the relative densitometric values, expressed as fold-over siCtrl sample. Similar results were obtained in at least 3 independent experiments. **C-D)** E2F inhibitor HLM (E2Fi) dose-dependently downregulates mRNA levels of E2F targets and reduces protein

levels of E2F1 and E2F2. **C)** RT-qPCR analysis of *BRCA1*, *CDC6*, *E2F1* and *E2F2* in PC3 cells 24h after treatment with E2Fi (20, 40 μM). *L19* was used as normalization control. Results are expressed as fold-over samples treated with vehicle (mean ± SD) from one experiment done in triplicate. **D)** Representative Western blot analysis of E2F1 and E2F2 in PC3 cells 24h after treatment with E2Fi (20 μM). Expression of HSP90 was used as loading control. Numbers below the bands correspond to the relative densitometric values, expressed as fold-over vehicle sample. Similar results were obtained in at least 3 independent experiments.

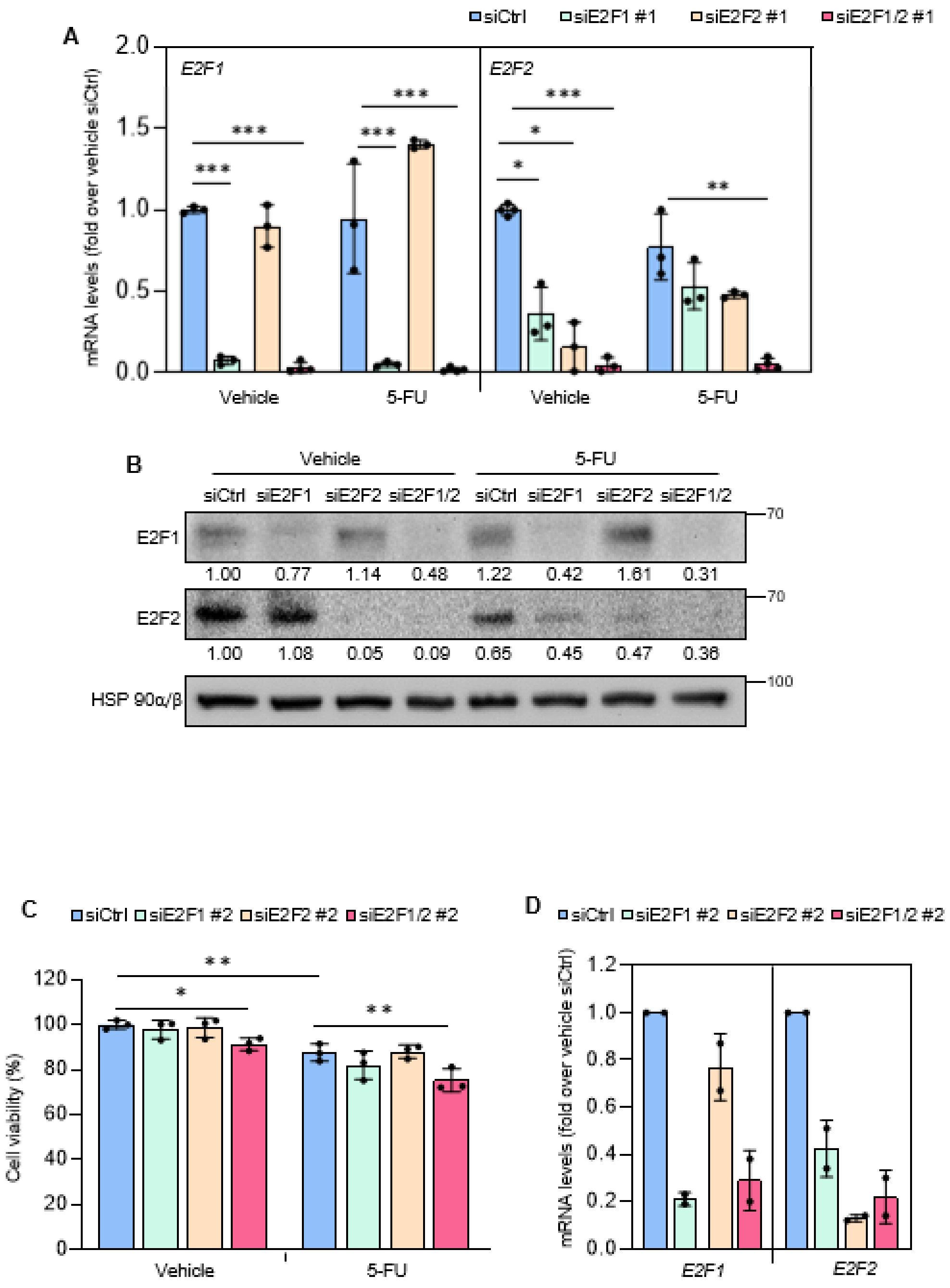

Supplementary Figure S3. **A)** Silencing efficiency of set #1 siRNAs for E2F1 and E2F2. RT- qPCR analysis of E2F1 and E2F2 in PC3 cells 96h after transfection with non-target control siRNAs (siCtrl), siRNAs specific for E2F1 (siE2F1#1), for E2F2 (siE2F2#1) or with their combination (siE2F1/2#1) and treatment with 5-FU (5 µM) for the last 72h. *L19* was used as normalization control. Results are expressed as fold-over vehicle siCtrl sample (mean ± SD) from 3 independent experiments. *** $p < 0.0001$, ** $p < 0.001$, * $p < 0.05$. **B)** Representative Western

blot analysis of E2F1 and E2F2 in extracts prepared as previously detailed. Expression of HSP90 was used as loading control. Numbers below the bands correspond to the relative densitometric values, expressed as fold-over vehicle siCtrl sample. Similar results were obtained in at least 3 independent experiments. **C)** PC3 cell viability 96h after transfection with non-target control siRNAs (siCtrl), with siRNAs specific for E2F1 (siE2F1 #2) or E2F2 (siE2F2 #2) or for E2F1 and E2F2 (siE2F1/2 #2) and treatment with 5-FU (5 μM) for the last 72h. Cellular viability was measured by FACS analysis after cell fixation and PI staining to detect DNA content and determined as the percentage of cells that were not in SubG0/G1 relative to siCtrl. Data shows the average ±SD from 3 independent experiments. *p<0.05. **D)** Silencing efficiency of set #2 siRNAs for E2F1 and E2F2. RT- qPCR analysis of E2F1 and E2F2 in PC3 cells 96h after transfection with non-target control siRNAs (siCtrl), siRNAs specific for E2F1 (siE2F1#2), for E2F2 (siE2F2#2) or with their combination (siE2F1/2#2). *L19* was used as normalization control. Results are expressed as fold-over siCtrl (mean ± SD) from 2 independent experiments.

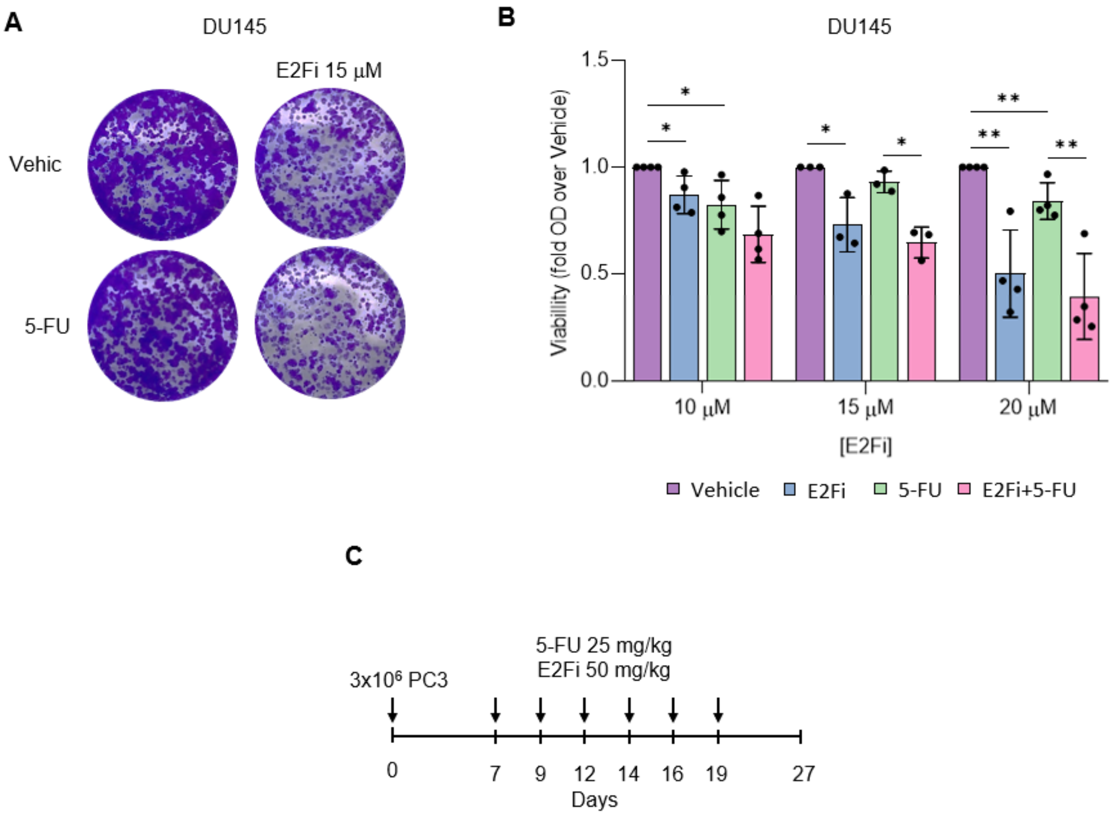

D

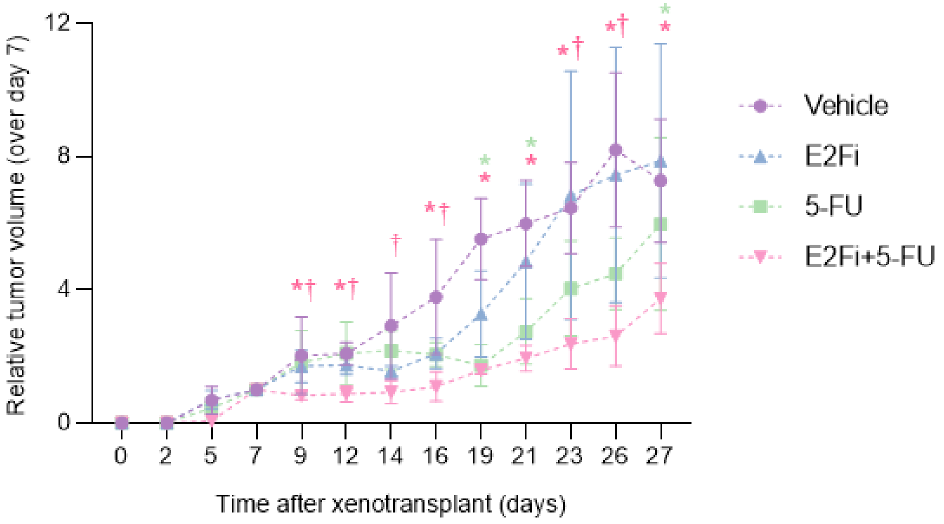

E

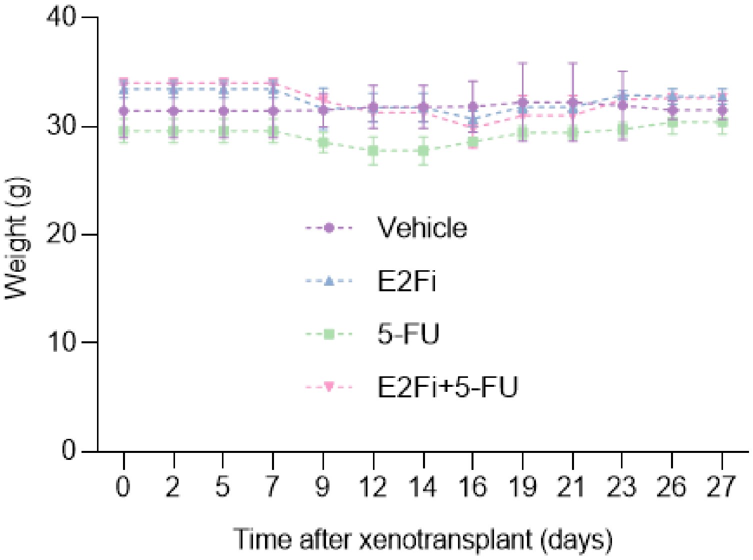

__**Supplementary Figure S4**__. **A)** Representative images of colony density in DU145 cells in each condition. DU145 cells were treated with 5FU (0.5 µM) and HLM (10, 15, 20 µM) alone or in combination. 72 hours later, drugs were washed out and cells were incubated with fresh media additional 10-12 days. Then, cells were fixed and stained with crystal violet. **B)** Quantification of CFA represented in panel A. Crystal violet was dissolved with acetic acid and the absorbance was measured by spectrophotometry. Relative cell viability was calculated by normalizing the absorbance of each condition using their vehicle controls. ** $p < 0.001$, * $p < 0.05$. **C)** PC3 xenograft growth after treatment with 5-FU and HLM. PC3 cells were injected subcutaneously into both flanks in 8 CD-1 nude mice. When tumor volume reached 65 mm^3, mice were divided

in 4 groups (vehicle, 5-FU, HLM and combination). 5-FU and HLM were administered IP every 2 to 3 days at 25 mg/kg and 50 mg/kg, respectively, along or in combination. **D)** Growth curves depict mean (± SD) of the relative tumor volume over respective volume at the initiation of the treatment. n=3-4 tumors per group. * p< 0.05 *vs.* vehicle, † p< 0.05 *vs.* 5-FU. **E)** Mouse weight in xenograft experiment. n=2 mice per group.

A

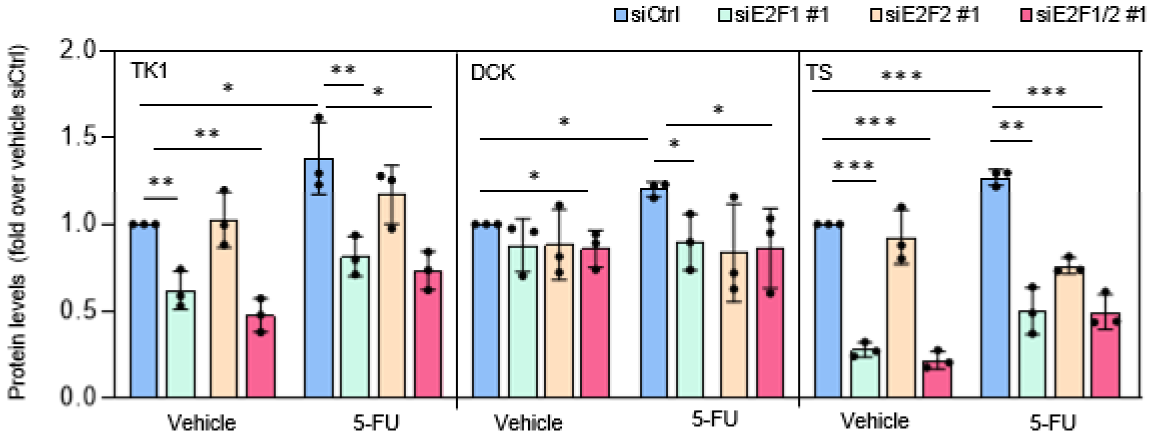

B

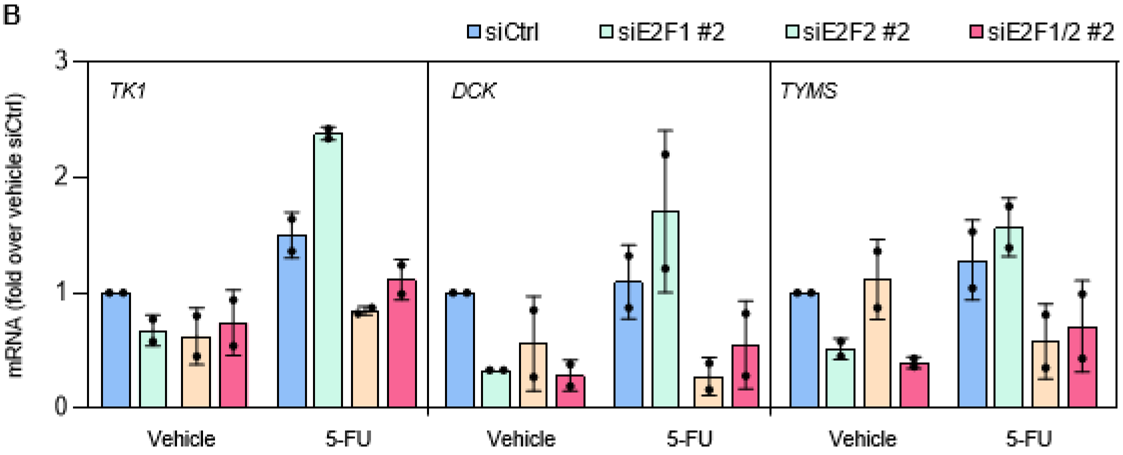

C

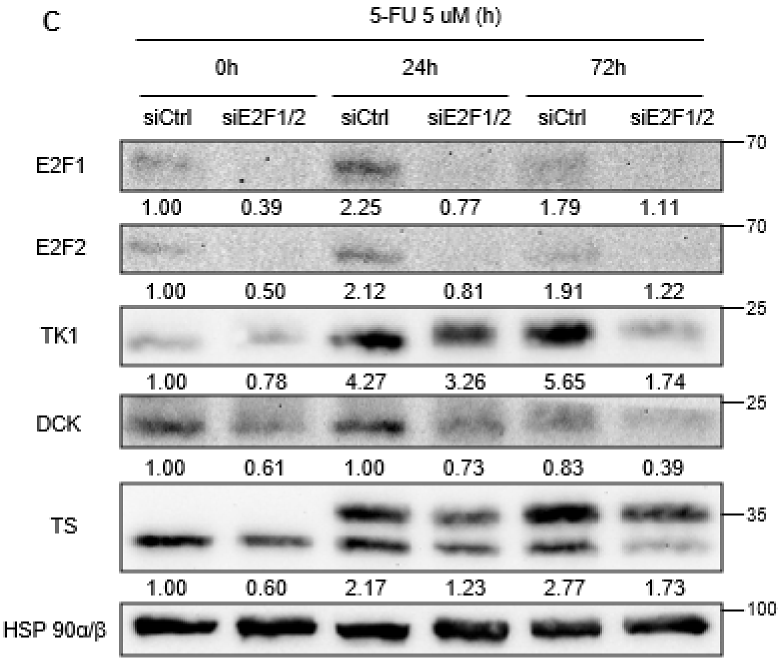

<u>Supplementary Figure S5.</u> **A)** Densitometric quantification of 3 independent experiments represented in the western blots shown in figure 3D. HSP90 was used as the normalization control. Results are expressed as fold-over vehicle siCtrl sample (mean ± SD) from 3 independent experiments. *** $p < 0.0001$, ** $p < 0.001$, * $p < 0.05$. **B-C)** Requirement of E2F1 and E2F2 for the expression of *TYMS*, *TK1* and *DCK* is observed using set#2 siRNA oligos in PC3 (**B**) and in DU145 mPCa cell line (**C**). **B)** RT-qPCR analysis of *TK1*, *DCK* and *TYMS* in PC3 cells 96h after transfection with non-target control siRNAs (siCtrl), siRNAs specific for E2F1 (siE2F1#2), for E2F2 (siE2F2#2) or with their combination (siE2F1/2#2) and treated for the last 72h with 5-FU (5 µM). *L19* was used as normalization control. Data are represented as fold-change relative to siCtrl treated with vehicle (-) from 2 independent experiments. **C)** Representative Western blot analysis of TK1, DCK and TS in extracts prepared from DU145 cells 96h after transfection with siCtrl, siE2F1#1, siE2F2#1, or siE2F1/2#1 and treated for the last 24 or 72h with 5-FU (5 µM). HSP90 was used as loading control. Numbers below the bands correspond to the relative densitometric values, expressed as fold-over 0h siCtrl sample. Similar results were obtained in at least 3 independent experiments.

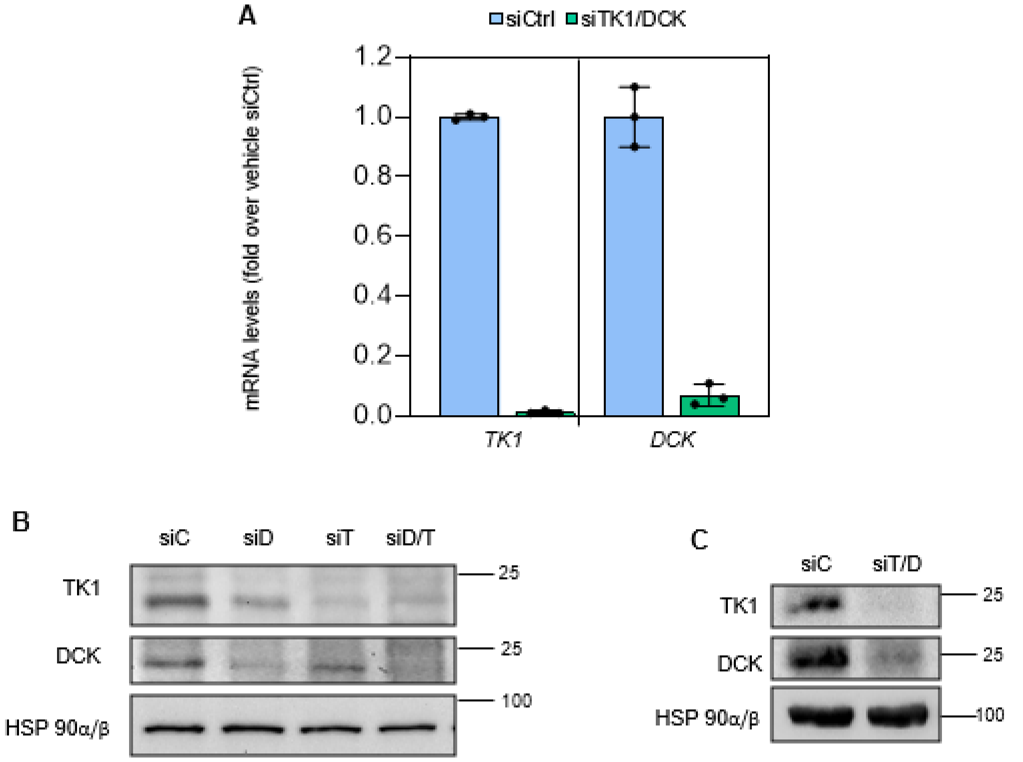

<u>Supplementary Figure S6</u>. **A)** RT- qPCR analysis of TK1 and DCK in PC3 cells 96h after transfection with non-target control siRNAs (siCtrl) or siRNAs specific for TK1 and DCK (siTK1/DCK#1). *L19* was used as normalization control. Results are expressed as fold-over vehicle siCtrl sample (mean ± SD) from one experiment done in triplicate. **B-C)** Representative Western blot analysis of TK1 and DCK 72h after transfection with with siCtrl (siC), siDCK (siD), siTK1 (siT), or siDCK/TK1 (siD/T). Expression of HSP90 was used as loading control.

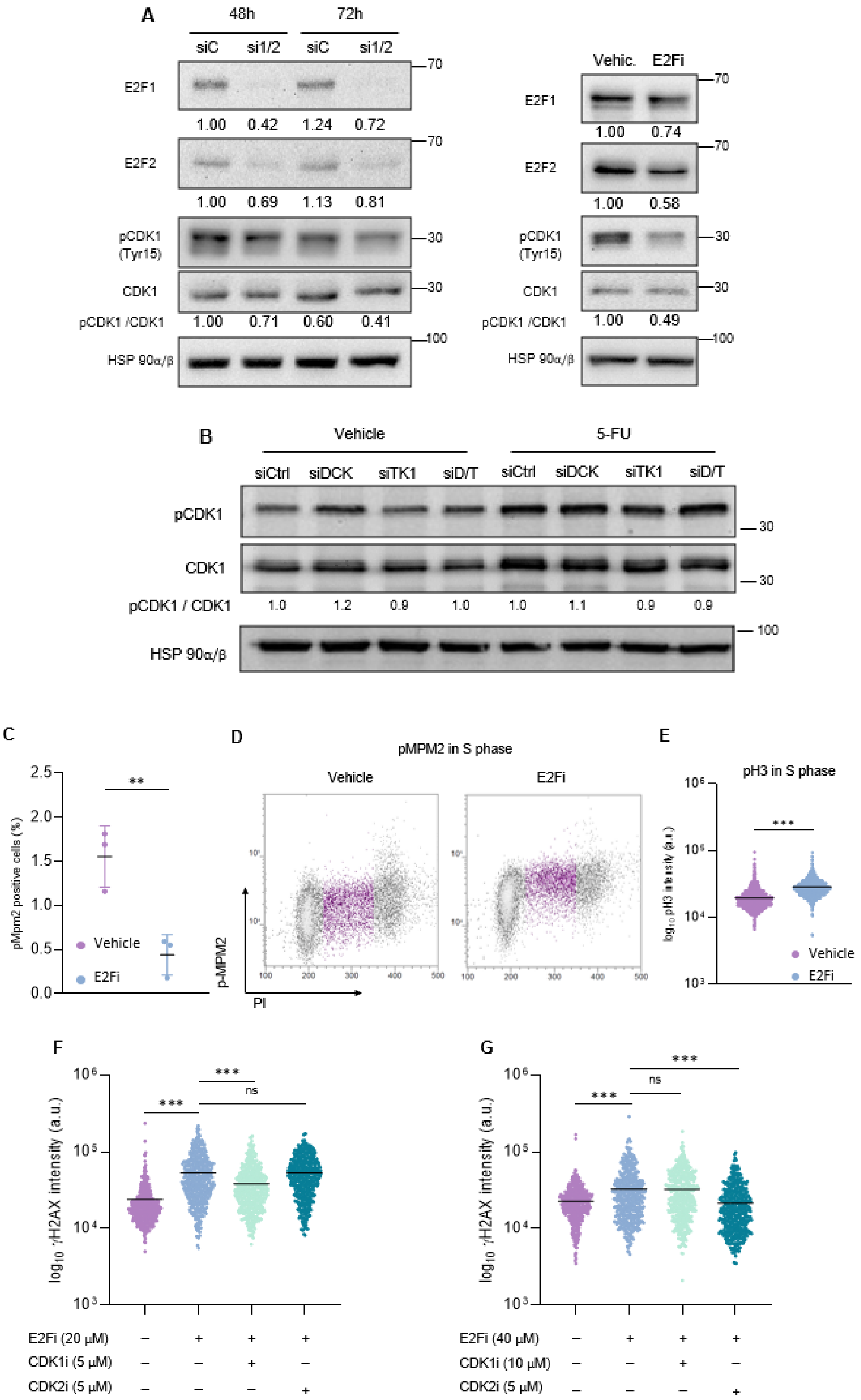
A
48h
72h
siC
si1/2
siC
si1/2
E2F1
1.00 0.42 1.24 0.72
E2F2
1.00 0.69 1.13 0.81
pCDK1 (Tyr15)
CDK1
pCDK1 /CDK1
1.00 0.71 0.60 0.41
HSP 90α/β
70
30
100
Vehic.
E2Fi
1.00 0.74
1.00 0.58
1.00 0.49
B
Vehicle
5-FU
siCtrl siDCK siTK1 siD/T siCtrl siDCK siTK1 siD/T
pCDK1
CDK1
pCDK1 / CDK1
1.0 1.2 0.9 1.0 1.0 1.1 0.9 0.9
HSP 90α/β
C
pMpm2 positive cells (%)
Vehicle
E2Fi
**
D
pMPM2 in S phase
Vehicle
E2Fi
p-MPM2
PI
E
pH3 in S phase
log10 pH3 intensity (a.u.)
***
Vehicle
E2Fi
F
log10 γH2AX intensity (a.u.)
***
ns
E2Fi (20 μM)
CDK1i (5 μM)
CDK2i (5 μM)
G
E2Fi (40 μM)
CDK1i (10 μM)
CDK2i (5 μM)

Supplementary Figure S7. E2F1/2 knockdown or E2Fi increases CDK1 activity in DU145 cells, and inhibition of CDK2 with CAS 222035-13-4 (CDK2i) prevents E2Fi-induced DNA damage in DU145 cells but not in PC3 cells. **A)** Representative Western blot analysis of CDK1 phosphorylated in Tyr 15 (pCDK1) and total CDK1 in extracts prepared from DU145 cells 48 or 72h after transfection with siCtrl or siE2F1/2#1 (left panel) or treated with E2Fi (40 μM) for 8h (right panel). HSP90 was used as loading control. Densitometric values were used to calculate pCDK1 / CDK1 relative levels, expressed as fold-over vehicle siCtrl or vehicle samples. **B)** Representative Western blot analysis of CDK1 phosphorylated in Tyr 15 (pCDK1) and total CDK1 in extracts prepared from PC3 cells 96h after transfection with siCtrl, siDCK, siTK1, or siDCK/TK1 and 5-FU or vehicle treatment during the last 72h. HSP90 was used as loading control. Densitometric values were used to calculate pCDK1 / CDK1 relative levels. **C)** Percentage of PC3 pMPM2-positive cells with 4C DNA content 24h after treatment with E2Fi (20 μM). **$p<0.005$. **D)** Representative dot plots of pMPM2/PI double staining after treatment with E2Fi (20 μM). In purple is highlighted the S-phase cell population gated for the analysis of pMPM2 intensity shown in Fig. 6F. **E)** Intensity of pH3 signal in cells in S phase 24h after treatment with E2Fi (20 μM). Data are expressed as Log10 intensity. Horizontal bars mark mean intensity. Data of 1000 cells from one representative experiment of 3 independent experiments are shown. **F-G)** Intensity of γH2AX signal in cells in S phase after the indicated treatments in PC3 (left panel and DU145 (right panel) cell lines. Data are expressed as Log10 intensity. Horizontal bars mark mean intensity. Data of 500 cells from one experiment representative of 3 independent experiments are shown. ***$p<0.0001$. ns= non-significant.

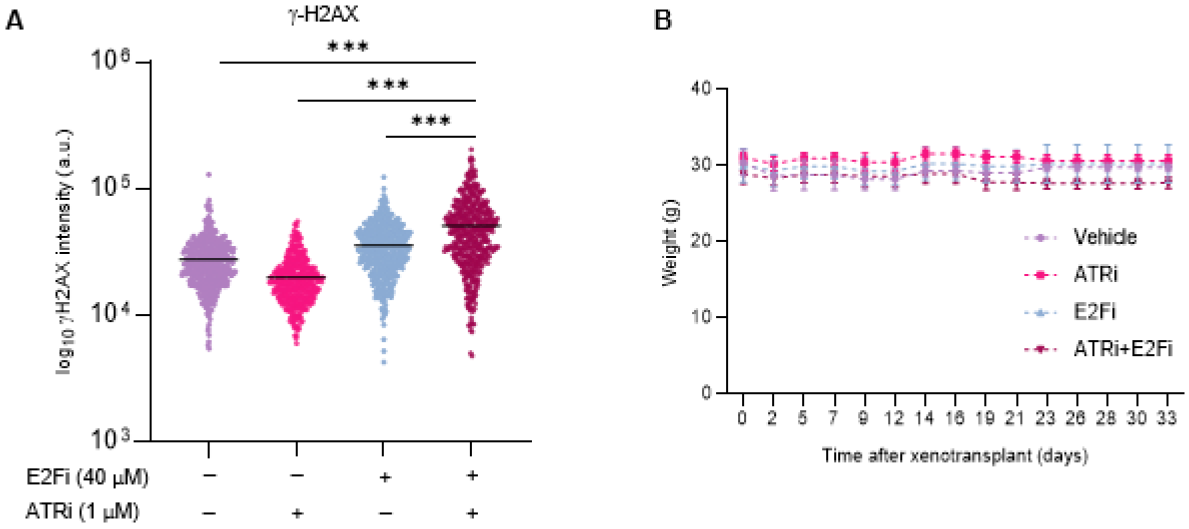

Supplementary Figure S8. **A)** Intensity of γH2AX signal in DU145 cells in S phase 4h after the indicated treatments. Data of 500 cells from one experiment representative of 3 independent experiments are shown. Data are expressed as Log10 intensity. Horizontal bars mark median intensity. ***p<0.0001. **B)** Mouse weight in xenograft experiment shown in figure 7C-E. n=3-5 mice per group.
